# Bacterial, Archaeal, and Eukaryotic Diversity across Distinct Microhabitats in an Acid Mine Drainage

**DOI:** 10.3389/fmicb.2017.01756

**Published:** 2017-09-12

**Authors:** Victoria Mesa, Jose L. R. Gallego, Ricardo González-Gil, Béatrice Lauga, Jesús Sánchez, Celia Méndez-García, Ana I. Peláez

**Affiliations:** ^1^Department of Functional Biology – IUBA, University of Oviedo Oviedo, Spain; ^2^Vedas Research and Innovation, Vedas CII Medellín, Colombia; ^3^Department of Mining Exploitation and Prospecting – IUBA, University of Oviedo Mieres, Spain; ^4^Department of Biology of Organisms and Systems – University of Oviedo Oviedo, Spain; ^5^Equipe Environnement et Microbiologie, CNRS/Université de Pau et des Pays de l’Adour, Institut des Sciences Analytiques et de Physico-chimie pour l’Environnement et les Matériaux, UMR5254 Pau, France; ^6^Carl R. Woese Institute for Genomic Biology, Urbana IL, United States

**Keywords:** acid mine drainage, Bacteria, Archaea, Eukarya, sediment, ore, biofilm, stalactite

## Abstract

Acid mine drainages are characterized by their low pH and the presence of dissolved toxic metallic species. Microorganisms survive in different microhabitats within the ecosystem, namely water, sediments, and biofilms. In this report, we surveyed the microbial diversity within all domains of life in the different microhabitats at Los Rueldos abandoned mercury underground mine (NW Spain), and predicted bacterial function based on community composition. Sediment samples contained higher proportions of soil bacteria (AD3, Acidobacteria), as well as Crenarchaeota and Methanomassiliicoccaceae archaea. Oxic and hypoxic biofilm samples were enriched in bacterial iron oxidizers from the genus *Leptospirillum*, order Acidithiobacillales, class Betaproteobacteria, and archaea from the class Thermoplasmata. Water samples were enriched in Cyanobacteria and Thermoplasmata archaea at a 3–98% of the sunlight influence, whilst Betaproteobacteria, Thermoplasmata archaea, and Micrarchaea dominated in acid water collected in total darkness. Stalactites hanging from the Fe-rich mine ceiling were dominated by the neutrophilic iron oxidizer *Gallionella* and other lineages that were absent in the rest of the microhabitats (e.g., Chlorobi, Chloroflexi). Eukaryotes were detected in biofilms and open-air water samples, and belonged mainly to clades SAR (Alveolata and Stramenopiles), and Opisthokonta (Fungi). Oxic and hypoxic biofilms displayed higher proportions of ciliates (*Gonostomum, Oxytricha*), whereas water samples were enriched in fungi (*Paramicrosporidium* and unknown microbial Helotiales). Predicted function through bacterial community composition suggested adaptive evolutive convergence of function in heterogeneous communities. Our study showcases a broad description of the microbial diversity across different microhabitats in the same environment and expands the knowledge on the diversity of microbial eukaryotes in AMD habitats.

## Introduction

Acid mine drainages (AMD) form when sulfide minerals (e.g., pyrite and FeS_2_) are exposed to oxygen and water during metal ore mining (Nordstrom and Alpers, 1999). Pyrite dissolution is enhanced by the activity of autochthonous iron oxidizing bacteria and archaea, which contribute to the formation of acidic and metal-rich solutions that drain from mine wastes and mining activities, generating AMDs (Silverman and Ehrlich, 1964; Baker and Banfield, 2003). Low pH increases the solubility of certain metallic species present in secondary minerals, thereby increasing the metal load of the drainage (Larios et al., 2012). Communities of autotrophic/heterotrophic bacteria and archaea thrive in these conditions, and ultimately control the cycling of biogeochemical elements Fe, S, C, N, and H in AMDs (Baker and Banfield, 2003).

The main environmental variables influencing distribution of microbial species in acidic mine outflows are pH, temperature, concentrations of dissolved metals, total organic carbon, and dissolved oxygen (Méndez-García et al., 2015). AMD systems constitute approachable models for microbial ecology analysis due to their relatively low microbial species richness and the existence of a tight coupling of biological and geochemical processes.

Los Rueldos Hg mine (Asturias, NW Spain) constitutes a recently explored example of an AMD formation (Méndez-García et al., 2014). The emplacement appears as a cave opened in a cinnabar (HgS)-rich mountain slope and was associated to the process of Hg recovery until its abandonment, more than 40 years ago. Los Rueldos displays a drainage of pH 2, with levels of arsenic and aluminum above the limits allowed by the Spanish legislation for direct discharge (Méndez-García et al., 2014). Its more distinctive characteristic, as compared to other AMD ecosystems, is the presence of massive streamer biofilms developing in shallower regions of a rather static drainage, whereas thick microbial biofilms thrive under hypoxic conditions in stagnant ponds across its course. While the microbial diversity and function of biofilms thriving at oxic and suboxic conditions have been investigated (Méndez-García et al., 2014), the microbial diversity, including microbial eukaryotes, across the identifiable microhabitats within the drainage remained unexplored.

The microbial ecology at discrete microhabitats within AMDs across the globe has been extensively reviewed recently (Méndez-García et al., 2015; Chen et al., 2016). Nevertheless, there are only few studies on the comparative microbial diversity across different microhabitats within the same AMD (Amaral-Zettler, 2012; Falagán et al., 2014; Jones et al., 2015). Thus, our knowledge on the microbial composition at different microhabitats in AMD systems has been inferred from discrete studies on different AMD microhabitats. For example, bacteria inhabiting thick biofilms differ from bacteria inhabiting thin biofilms and acid waters in distinct ecosystems (Bond et al., 2000a; Hallberg et al., 2006; Hao et al., 2010), and archaea diverge taxonomically in separate microhabitats from different AMDs (Baker and Banfield, 2003; Bruneel et al., 2008; Hao et al., 2010; Méndez-García et al., 2014).

Eukaryotes inhabiting AMD systems have been provided comparatively little attention, and knowledge about their involvement in the biogeochemical cycles in AMDs is still limited (Amaral-Zettler, 2012; Aguilera, 2013; Volant et al., 2016). The diversity of eukaryotic microorganisms inhabiting open air AMD systems includes microscopic algae, which are primary producers, protozoans (ciliates, flagellates, rotifers, amoebae), contributing to primary or secondary production, and fungi, which act as decomposers and contribute to carbon recycling (Méndez-García et al., 2015). Fungi and protists confer structure to the biofilms and impact the community composition by grazing on resident bacteria and archaea (Baker et al., 2004). In soils, protozoa can affect the structure of the bacterial communities (Rosenberg et al., 2009), or might impact their dispersal (Brock et al., 2011).

Acid mine drainages constitute the main source of pollution of fresh surface waters on Earth. These metal-rich mine outflows are highly toxic and, when mixed with groundwater, surface water, or soil, they become responsible for the contamination of drinking water, disruption of growth and reproduction of aquatic plants and animals, or corrosion of infrastructures. Most microbes thriving in these ecosystems obtain their energy through the oxidation of reduced metallic species and have potential for mineral bioleaching. As the generation of acidic leachates occurs during this process, a possible solution for the remediation of AMDs consists in preventing the oxic conditions that allow the activity of the microbes involved. Nevertheless, the selective pressures operating in these extreme environments (low pH, toxic concentration of metals) have equipped them with diverse adaptive mechanisms that make their biology a thrilling subject of study (Johnson and Hallberg, 2003).

The current report describes the diversity of the microorganisms pertaining to the Bacteria, Archaea, and Eukarya domains of life across distinct microhabitats (acid water, mineral fractions including ore and sediments, and different biofilms morphologies) in Los Rueldos AMD using low as well as high-throughput taxonomic profiling of phylogenetic markers (16S/18S rRNA genes). We further predicted microbial function by metagenomic reconstruction through 16S rRNA genes survey. With our scrutiny, we attempted to expand our knowledge on the microbial eukaryotes and bacteria/archaea present in unexplored microhabitats within Los Rueldos AMD. Thus, this extreme ecosystem represents an excellent natural laboratory in which complex questions about evolution and functionality of microbial communities could be investigated.

## Materials and Methods

### Samples Description and Measurement of Environmental Variables

**FIGURE 1:**
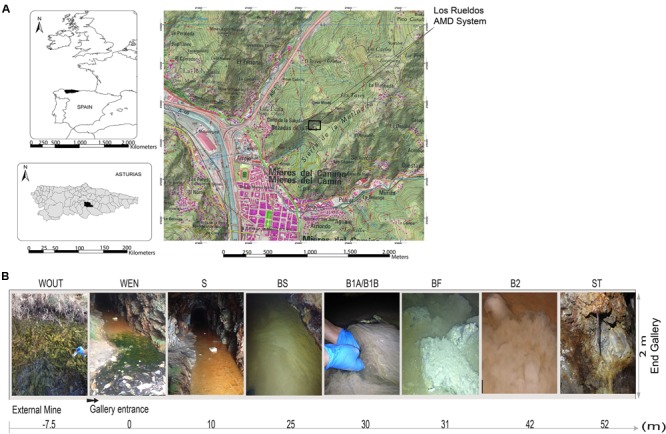
Maps of the Los Rueldos emplacement in Asturias (NW Spain) **(A)**. Depiction of the sampling locations along the AMD **(B)**.

The geological features and geochemistry of Los Rueldos mine in Asturias have been extensively described elsewhere (Méndez-García et al., 2014). AMD samples were aseptically collected in triplicates in March 2013 from 10 sampling points in sterile 50 mL tubes and kept at 4°C until being processed in the laboratory (within 2 h of collection). Samples included water (WOUT, WEN, and WIN), mineral fractions (sediment, S, and ore samples collected from stalactites, ST), and microbial biofilms (BS, BF, B1A, B1B, and B2; see **Table 1** for a description of samples) (**Figure 1** and **Table 1**).

**Table 1:** List of samples analyzed in this study.

Type of sample	Sample	Description
Water	WOUT	Acid water from pond outside of the gallery
	WEN	Acid water from pond at the entrance of the gallery
	WIN	Acid water filtered through a 0.22 μm pore size membrane filter. Analysis on the retained fraction.
Sediment	S	AMD bed sediment
Stalactite	ST	Surface of stalactites generated at the cave ceiling
Biofilm	BF	Subaerial biofilm at the interface rock/acid water
	BS	Biofilm at the interface sediment/water at a depth of 15 cm
	B1A/B1B	Stratified streamer with uppermost (A) and lowermost (B) strata
	B2	Submerged microbial mat at drainage depth ∼50 cm

Conductivity (mS cm^-1^), temperature (°C), redox potential (mV), and pH were measured at three discrete points in each sampling location using a portable multi-parameter probe (Multi 340i, WTW, Germany). Luminance (Lx) was determined using Lunasix 3s (Gossen).

Morphological information about the eukaryotes present in the WOUT sample was obtained by phase contrast microscopy, using a Nikon Eclipse E-200 microscope coupled to a Nikon Cool-Pix 990 digital camera.

### Denaturing Gradient Gel Electrophoresis (DGGE)

Sequence-specific separation by DGGE (Muyzer et al., 1993) was performed in B1A, B1B, and B2 samples collected during the period 2007–2012 using 300–500 ng of pooled PCR products obtained by the amplification of the V3–V5 variable regions of the 16S rRNA gene with primer pairs 341fGC/907R (Muyzer, 1999; Muyzer et al., 1993) for Bacteria and 344fGC/915R (Stahl and Amann, 1991 and Raskin et al., 1995) for Archaea. An annealing temperature of 55°C provided the best results for all group amplifications. DGGE ran in a DCode^TM^ Universal Mutation Detection System (Bio-Rad) in Tris-acetate-ethylenediaminetetraacetic acid (TAE) buffer using 6% (w/v) poly-acrylamide 1 mm thick gels (40% Acrylamide/Bis Solution 37.5:1, Bio-Rad). Solutions of 0, 30, and 70% of denaturing agents [100% denaturing solution with 7 M urea and 40% formamide (v/v)] were used to build the gradient. The electrophoresis coursed at 70 V for 16 h at 60°C. A pre-running step for 5 min at 110 V was included to avoid dispersion of the sample. The gel was stained with SYBR Gold Nucleic Acid Gel Stain (Invitrogen, Life Technologies^TM^, United States) and scanned with a Kodak Gel Logic 200 Imaging System (Kodak, United States). Band profiles were analyzed using Phoretix 1D Pro software (TotalLab Ltd., United Kingdom) and clustered by UPGMA (Unweighted Pair Group Method with Arithmetic mean) analysis.

### Large-Scale Parallel Pyrosequencing of 16S and 18S rRNA Genes

Microbial community composition within the domains Bacteria, Archaea, and Eukarya was addressed by high-throughput 454 pyrosequencing. DNA extraction was carried out from three replicate samples collected at each sampling point. The three replicates were then composted together before statistical analysis. Bacterial V1–V3 region of the 16S rRNA gene was amplified using the primer set 27F (5′-AGAGTTTGATCCTGGCTCAG-3′) and 338R (5′-TGCTGCCTCCCGTAGGAGT-3′) (Hongoh et al., 2003; Fierer et al., 2008); Archaeal V3–V5 region of the 16S rRNA gene was amplified using Arch349F (5′-CCCTACGGGGTGCASCAG-3′) and Arch806R (5′-GGACTACVSGGGTATCTAAT-3′) (Hugoni et al., 2013), and Eukaryotic V9 region of the 18S rRNA using 1380F (5′-CCCTGCCHTTTGTACACAC-3′) and 1510R (5′-CCTTCYGCAGGTTCACCTAC-3′) (Amaral-Zettler et al., 2009). Primers were bound to 10-nucleotide multiplexing tags and to the 454 FLX sequencing adaptors A (5′-CCATCTCATCCCTGCGTGTCTCCGACTCAG-3′) and B (5′-CCTATCCCCTGTGTGCCTTGGCAGTCTCAG-3′) in the forward and reverse regions, respectively. PCR reactions were performed using AmpliTaq Gold 360 Master Mix (Applied Biosystems, United States) following the instructions provided by the manufacturer. Products obtained from separate reactions were pooled and purified using Gel Band Purification Columns (GE Healthcare, United Kingdom), and DNA concentration was determined using Qubit dsDNA assay kit (Invitrogen). Sequencing was carried out on a 454 Life Sciences Genome Sequencer FLX (Roche 454 Life Sciences, Branford, CT, United States) at Macrogen Inc. (Seoul, South Korea). The raw reads have been deposited into the NCBI short-reads archive database (BioProject Accession: PRJNA391850).

### Bioinformatic Analyses of High Throughput Sequencing Data

16S/18S rRNA gene amplicon sequences were analyzed using QIIME version 1.9 (Caporaso et al., 2010a). Reads were quality checked (minimum length was set to 200 bp, ambiguous bases and mismatches in primer sequences were not allowed, minimum quality score was 25) and assigned to samples based on nucleotide barcodes. Quality-controlled sequences were denoized using the QIIME denoiser. Chimeras were predicted with ChimeraSlayer and removed prior to downstream analyses. Chimera-free sequences were clustered into operational taxonomic units (OTUs) defined by a 97% sequence similarity cutoff using UCLUST (Edgar, 2010). Representative sequences from each OTU were aligned using PyNAST (Caporaso et al., 2010b) and singletons were excluded from the analysis. Taxonomic annotation was performed through comparison against the Greengenes database (release May, 2013) for 16S rRNA gene amplicon data, and with the SILVA non-redundant database, release 128 (September, 2016) for the 18S rRNA gene amplicon data.

### Statistical Analyses of High Throughput Sequencing Results

QIIME data were imported into the R version 3.2.4 (R Development Core Team, 2016). Analyses and subsequent visualizations were performed using functions from ggplot2 version 1.0.0 (Wickham, 2009). Alpha diversity inferences included determination of observed number of OTUs per sample, species richness estimation (Chao1), and calculation of diversity indices (Shannon and Simpson) using the package {phyloseq}, version 1.7.12 (McMurdie and Holmes, 2013). Rarefaction curves were generated using the {vegan} package (Oksanen et al., 2016). Beta diversity was evaluated through principal coordinate analysis (PCoA) of weighted UniFrac distances obtained through QIIME (Lozupone and Knight, 2005) to visualize similarities in the microbial composition. Canonical correspondence analysis (CCA) was performed to explore the environmental factors that had the most significant influence on the microbial community structure. We used the adonis() function from the vegan library, which fits linear models to distance matrices and uses a permutation test with Pseudo F-ratios.

### Prediction of Functional Content from Marker Gene Survey and Statistical Analysis of PICRUSt Results

Bacterial function was inferred using the 16S rRNA gene high throughput sequencing data. QIIME biological observation matrices were imported into PICRUSt (Langille et al., 2013), and metagenomes inferred using the proposed pipeline. PICRUSt uses marker gene data to query a reference database for the closest reference genome available. Genomic-driven inference of function is then used to predict gene families, which are therefore combined to estimate the composite metagenome (Langille et al., 2013). Briefly, a PICRUSt-compatible OTU table was constructed in QIIME. Normalization by 16S rRNA copy number per OTU was performed with the normalize_by_copy_number.py script, followed by metagenome functional prediction for each sample (predict_metagenomes.py and categorize_by_function.py). The accuracy for the predicted metagenome was tested through the Nearest Sequenced Taxon Index (NSTI), reflecting the presence of reference genomes that are closely related to the samples in analysis.

PICRUSt results were imported into R (R Development Core Team, 2016). Probability distribution of data was determined using the function *descdist* from the {fitdistrplus} package and uniformity of variance was tested with the Levene test (leveneTest{car}). Differences between experimental groups were evaluated using the Kruskal–Wallis test for continuous variables (kruskal.test{stats}). A *p*-value of less than 0.05 was considered to indicate statistical significance.

### SSU rRNA Gene (18S) Clone Libraries Construction, Sequencing, and Gene Phylogeny Reconstruction

The diversity of eukaryotes was analyzed in parallel through full length 18S rRNA gene clone library construction. DNA from water, mineral fractions, and biofilms samples was extracted using the PowerSoil DNA Isolation Kit (MoBio, United States) according to manufacturer’s recommendations. The 18S rRNA gene was amplified employing the primer set EK-42F (5′-CTCAARGAYTAAGCCATGCA-3′)/EK-1498R (5′-CACCTACGGAAACCTTGTTA-3′) (Marande et al., 2009). PCR amplicons were generated using AmpliTaq Gold 360 (Applied Biosystems, Life Technologies^TM^, United States) following the instructions provided by the manufacturer. The amplifications were performed in a MJ Mini Thermal Cycler (Bio-Rad, United States) with an annealing temperature of 55°C for 35 cycles. The PCR reactions obtained from separate reactions were pooled and purified using the Illustra GFX purification kit (GE Healthcare, United Kingdom). PCR products were cloned into pGEM-T Easy Vector System I (Promega, United States) following the manufacturer’s instructions. The ligation mixtures were then transformed into *Escherichia coli* competent JM109 cells (Promega Corporation). Transformant colonies were screened via PCR amplification of the inserts with flanking vector primers (M13F 5′-GTTTTCCCAGTCACGAC-3′; M13R 5′-GAAACAGCTATGACCATG-3′), and amplicons were sequenced according to the protocol of the BigDye Terminator v3.1 sequencing kit (Applied Biosystems) and subjected to capillary electrophoresis in an ABI PRISM 3130xl Genetic Analyzer (Applied Biosystems).

Sanger electropherograms were corrected and checked for chimera presence using the USEARCH (Edgar, 2010) using as reference the SILVA database (release 123_1) (Pruesse et al., 2007). 18S rRNA gene sequences were automatically aligned using the SINA aligner against the SILVA SSURef_123_1 reference alignment (Pruesse et al., 2012). The resulting alignment was manually examined to correct erroneously situated bases with ARB (Ludwig, 2004). A maximum likelihood phylogeny was constructed using general time reversible distances to define the nucleotide substitution model with RAxML (Stamatakis, 2014).

## Results

### Environmental Conditions across Collection Sites

**FIGURE 2:**
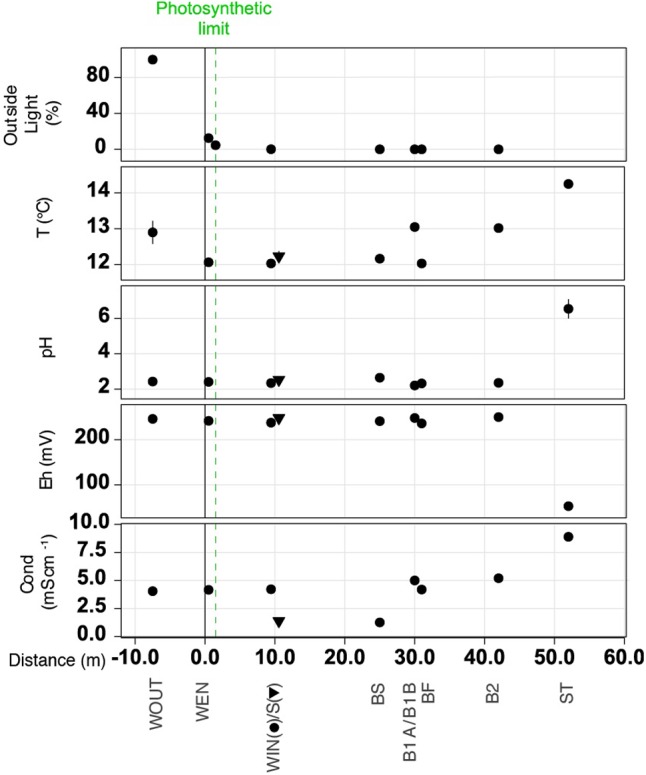
Spatial variation of geochemical variables [conductivity, Cond.; redox potential, Eh; pH; T, °C; and outside light (%)] of the AMD samples across collection sites. Symbols (dots or triangles) and vertical error bars represent the mean of the three measurements at each sampling location and their corresponding standard errors, respectively.

The AMD samples were characterized by a low pH (2 ± 0.95, mean ± SD), and a mean (±SD) temperature, redox potential, and conductivity of 12.49 ± 4.98°C, 232 ± 114 mV, and 4.38 ± 2.23 mS cm^-1^, respectively. An exception to this homogeneity was the stalactite (ST) sample, in which the dripping water showed a higher pH (6.5 ± 0.94), higher temperature (14.25°C), a lower redox potential (53 mV), and a higher conductivity (8.89 mS cm^-1^). The samples WOUT and WEN were located under the influence of the sunlight, receiving 98 and 3% of total irradiance, respectively. The rest of samples (WIN, S, BS, BF, B1A, B1B, and B2) were collected in total darkness (**Figure 2**).

### Microbial Diversity of Bacteria, Archaea, and Eukaryotes in the Different Microhabitats of Los Rueldos AMD

Bacterial 16S rRNA genes were successfully amplified from all samples, whereas archaeal 16S rRNA amplicons were obtained only from the samples WOUT, WEN, WIN, S, BS, and ST. Our research team had previously explored the bacterial and archaeal diversity in B1A, B1B, and B2 biofilms present in the drainage (Méndez-García et al., 2014). These data were used for comparative purposes in the present work due to the existence of very stable populations across time, as demonstrated through denaturing gradient gel electrophoresis (DGGE) analysis in B1AB and B2 throughout a 5-year period (Supplementary Figure S1). Eukaryotic 18S rRNA genes were successfully amplified from samples WOUT, WEN, and the streamer B1A.

**FIGURE 3:**
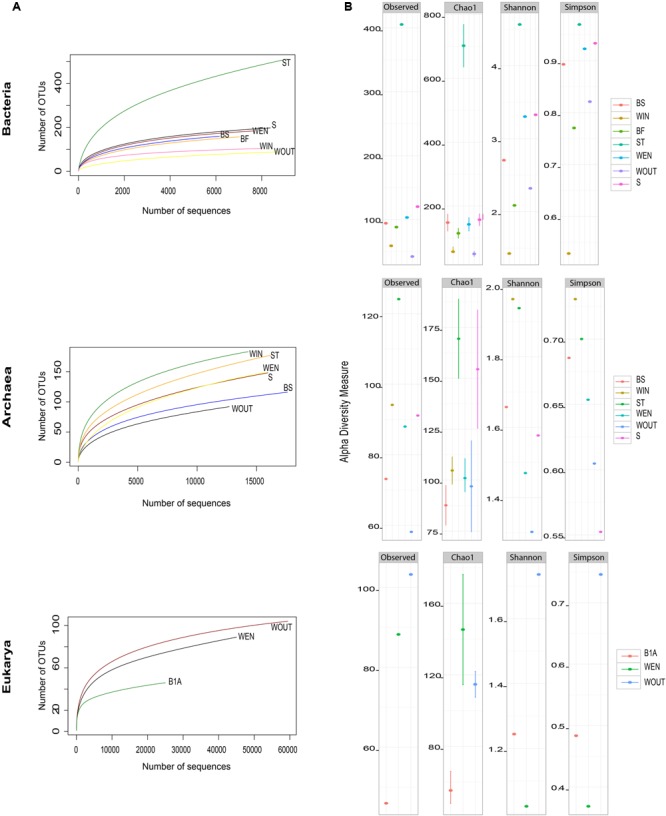
**(A)** Rarefaction curves at a 3% of dissimilarity cut-off among sequences. **(B)** Alpha diversity metrics (observed species, Chao1, Shannon, and Simpson indices) for Bacteria, Archaea, and Eukarya in Los Rueldos AMD samples.

The raw sequence data of the samples consisted of 456,928 (155,513 bacterial, 140,769 archaeal, and 160,646 eukaryotic reads), which were ultimately clustered into 844 bacterial, 535 archaeal, and 176 eukaryotic OTUs. Rarefaction curves showed that the bacterial diversity was recovered satisfactorily in all samples, whereas a more extensive sequencing effort would have been needed for total recovery of the archaeal and eukaryotic diversity (**Figure 3A**). The stalactite sample, ST displayed the highest bacterial diversity among all samples (metrics observed OTUs, Chao1, and Shannon and Simpson indices were 410, 700, 4.8, and 0.97, respectively). The samples showing a higher archaeal diversity were ST (observed OTUs, and Chao1, Shannon, and Simpson indices were 125, 173, 1.94, and 0.71, respectively) and WIN (Shannon and Simpson indices were 1.97 and 0.78, respectively). The highest diversity of eukaryotes was estimated for the water in the pond outside of the gallery (WOUT), which received the major influence of the sunlight (observed OTUs, and indices Chao1, Shannon and Simpson were 105, 119, 1.78, and 0.78, respectively) (**Figure 3B**).

#### Diversity of Bacteria

##### Bacterial phyla and classes

Proteobacteria was the most widely detected phylum (accounting for 51.9% of the total reads), followed by Cyanobacteria (12.4%, present only in WOUT and WEN), Nitrospirae (10%), Actinobacteria (7.2%), TM7 (hereinafter referred to as *Candidatus* Saccharibacteria, Albertsen et al., 2013) (4.2%), AD3 (2.7%), Acidobacteria (2.6%), and Firmicutes (0.64%).

**FIGURE 4:**
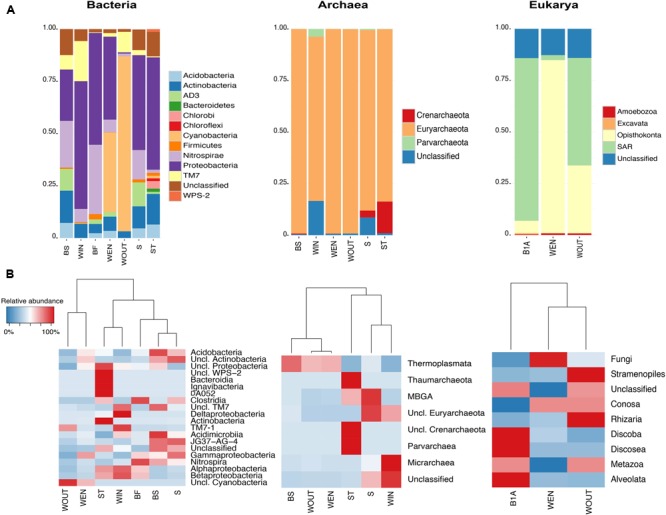
Taxonomy profiles displaying the microbial diversity in the AMD samples, revealed by high throughput sequencing of the 16S/18S rRNA genes. **(A)** Bar plots displaying the relative abundances at the phylum level within Bacteria, Archaea, and Eukarya. **(B)** Heatmaps showing the main classes detected within each phyla.

Bacterial taxa with relative abundances higher than 1% affiliated within the phyla Actinobacteria, Cyanobacteria, Nitrospirae, Proteobacteria, and *Ca*. Saccharibacteria in water samples (WOUT, WEN, WIN); within the Acidobacteria, Actinobacteria, AD3, Firmicutes, Nitrospirae, Proteobacteria, and *Ca*. Saccharibacteria in sediment (S); within Acidobacteria, Actinobacteria, AD3, Firmicutes, Nitrospirae, and Proteobacteria in the subaerial biofilm (BF); and within Acidobacteria, Actinobacteria, AD3, Bacteroidetes, Chlorobi, Chloroflexi, Cyanobacteria, Firmicutes, Nitrospirae, Proteobacteria, and WPS-2 in the surface of the stalactite (ST) (Bacteria, **Figure 4A**). A fraction of the sequences could not be assigned to any taxa at the phylum level and remained unclassified (1.3–5.6% in water samples, 9.8% in sediment sample, 12.4% in BS sample, 1.5% in the subaerial biofilm sample, and 11.6% in the stalactite). Proteobacteria dominated in all samples (61.1% WIN, 53.4% BF, 53.3% ST, 45.2% S, 39.6% WEN, and 24.5% BS), except in the acid water sample collected outside of the gallery (WOUT), where Cyanobacteria were predominant (Bacteria, **Figure 4A**).

Main bacterial classes detected in the outside water sample (WOUT) were unclassified Cyanobacteria (82.7%) and *Ca*. Saccharibacteria from an undetermined class (8.7%) (Bacteria, **Figure 4B**); classes detected in the water sample from the pond at the entrance of the gallery (WEN) were unclassified Cyanobacteria (37.9%), Gammaproteobacteria (29.8%), and Alphaproteobacteria (9.2%); the water inside of the gallery (WIN) contained higher proportions of Alpha-, Beta- and Deltaproteobacteria (51.7, 50.5, and 6.3%, respectively). Acidobacteria were predominant in the sediment and biofilm at the interface sediment/water, as compared to the other samples (7.2% in BS and 4.3% in S). BS was enriched in Nitrospira (22.3%), whereas S displayed a higher percentage of Gammaproteobacteria (36.1%). The subaerial biofilm contained distinctively more Nitrospira (33.1%), and the stalactite was enriched in Actinobacteria (13.6%), Ignavibacteria (3.4%), Bacteroidia (1.3%), and Alpha-, Beta-, and Gammaproteobacteria (41.2, 34.2, and 10.3%, respectively) (Bacteria, **Figure 4B**).

##### Genera and rare taxa

Dominant bacterial genera included *Acidimicrobium* (7% WIN, 3.15% WOUT, 3.35% BF, 12.15% BS, 3.85% WEN, and 5.4% S); *Leptospirillum* (35.9% BF, 23.55% BS, 14.8% S, 6.45% WIN, 6.45% WEN, 1.35% WOUT, and 0.2% ST); *Acidiphilium* (0.35% WEN, 0.2% BF, 0.1% S, and 0.1% WOUT); *Acidobacterium* (6.8% BS, 4% S, 3.2% WEN, 2.25% BF, and 2% ST); *Thiomonas* (6.55% ST); *Gallionella* (13% ST); *Acidithiobacillus* (1.8% BF, 1.2% WIN, 1.1% S, and 0.2% WEN), and an undetermined genus belonging to the *Ca*. Saccharibacteria (18% WIN, 9.8% WOUT, 6.3% BS, 2.2% S, 1.7% WEN, 0.5% ST, and 0.3% BF). The sediment (S) was characterized by the presence of RCP1-48 Gammaproteobacteria, Xanthomonadaceae bacteria, *Leptospirillum*, and AD3 bacteria; the stalactite (ST) was dominated by *Gallionella*, uncharacterized Actinomycetales (Actinobacteria), and Xanthomonadaceae; *Leptospirillum*, unknown Betaproteobacteria, Xanthomonadaceae, and RCP1-48 bacteria predominated in the subaerial biofilm BF; *Leptospirillum*, RCP1-48, and AD3 bacteria dominated in BS, and unknown genera affiliating within the Acidobacteria, Acidimicrobiales, and *Ca*. Saccharibacteria were present in proportions above 6%; WEN and WOUT were dominated by Cyanobacteria. Uncharacterized *Ca*. Saccharibacteria and RCP1-48 bacteria were the third most abundant groups detected in WOUT and WEN; respectively. Filtered water (WIN) was characterized by the presence of Betaproteobacteria, unclassified *Ca*. Saccharibacteria, and *Leptospirillum* (Supplementary Table S1A).

Rare taxa were defined as representing 0.1–1% relative abundance based on 16S rRNA hypervariable region sequence counts (Hugoni et al., 2013). Phyla with abundances < 1% included Armatimonadetes (0.05%), Bacteroidetes (0.15%), Chlorobi (0.4%), Chloroflexi (0.1%), Gemmatimonadetes (0.04%), NC10 (0.01%), and OD1 (0.02%) in the stalactite. In the sediment, minority taxa included Planctomycetes (0.01%) and WSP2 (0.2%). Elusimicrobia (0.03%) were detected in ST, S, and WEN and TM6 (0.01%) were identified in WIN (Supplementary Table S1A).

#### Diversity of Archaea

##### Archaeal phyla and classes

Archaea affiliating within the Euryarchaeota were predominant in all samples (99.25% WEN, 99.2% BS, 99.1% WOUT, 87.6% S, 83.6% ST, and 79.6% WIN). Crenarchaeota were detected only in stalactites (15.4% ST), sediment (3.4% S), and in the subaerial biofilm (0.4% BS). *Ca.* Parvarchaeota were detected in WIN (3.8%) (Archaea, **Figure 4A**). Class Thermoplasmata was predominant in WEN (93.7%), WOUT (93.1%), and BS (99.1%). The acidic water (WIN) displayed a higher proportion of *Ca.* Micrarchaea (3.75%). A variable proportion of sequences remained unclassified (0.61–17.08% in water samples, 8.75% in sediment sample, 0.38% in BS sample, and 1% in the stalactite). Thaumarchaeota (13.25%), unclassified Crenarchaeota (0.3%), and *Ca.* Parvarchaeota (0.1%) were more abundant in the stalactite (ST). The sediment (S) was enriched in MBGA (3.35%) and unclassified Euryarchaeota (0.7%) (Archaea, **Figure 4B**).

##### Archaeal genera

Archaeal genera *Ferroplasma* (2.6% FW and 9.15% ST) and *Thermogymnomonas* (93% POUT, 72% S, 74% FW, 69% ST, 84% DZ, and 93% PIN) predominated in Los Rueldos samples (Supplementary Table S1B).

#### Diversity of Eukaryotes

Eukaryotes across different microhabitats in Los Rueldos AMD primarily belonged to the supergroup SAR (Stramenopiles + Alveolata + Rhizaria) and Opisthokonta taxa. SAR were detected in all samples, being dominant in B1A and WOUT (78.9 and 52.1%, respectively). The Stramenopiles (1.7% WEN and 50.7% WOUT) were Diatomea of the class Bacillariophyceae (1.7% in WEN and 35% in WOUT) and Chrysophyceae (15.7% WOUT). Alveolata included Ciliophora of the class Spirotrichea (78.7% B1A, 0.5% WOUT, and 0.1% WEN). Detected Rhizaria belonged to the Cercozoa (0.9% WOUT and 0.1% WEN) (Eukarya, **Figure 4A**).

Opisthokonta were present in all samples (83.7% WEN, 32.9% WOUT, and 6% B1A), and were mainly represented by the fungal groups Ascomycota (30.4% WOUT), Chytridiomycota (1.6% WOUT), Basidiomycota (0.3% WEN), LKM11 (78.9% WEN, 0.6% WOUT, 4.9% B1A), Nucletmycea (4.5% WEN, 0.1% WOUT, and 0.7% B1A), and LKM15 (0.1% WOUT and 0.2% B1A) (Eukarya, **Figure 4B**).

Taxa with abundances < 1% included representatives of the supergroups Amoebozoa (1.6%) and Excavata (0.7%). Unclassified sequences accounted for a 12.7–14.2% in all samples (Supplementary Table S1C).

Micrographs showing the morphology of the different eukaryotic species present in open-air water samples collected at Los Rueldos (filamentous algae, cyanobacteria, diatoms, and protozoans) are presented in Supplementary Figure S2.

**FIGURE 5:**
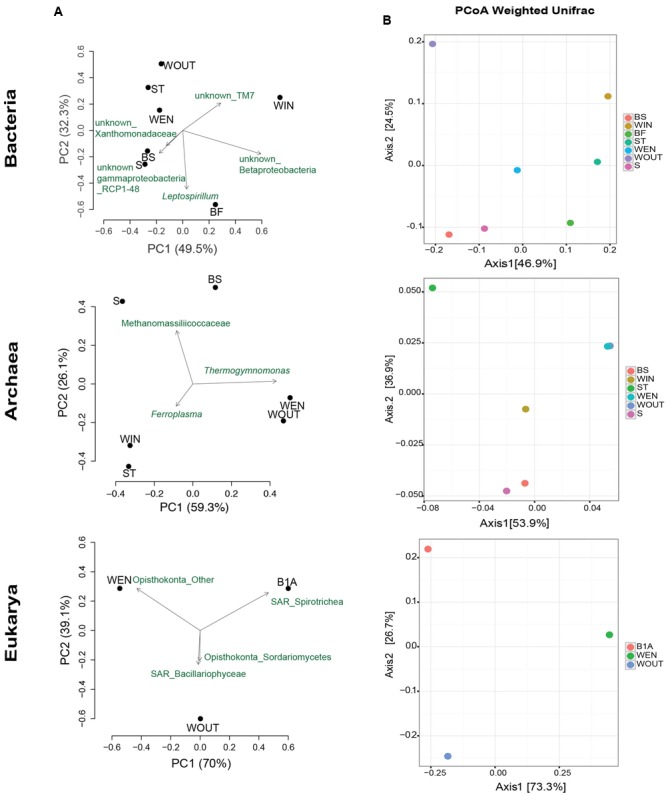
Principal coordinates analysis (PCoA) based on the weighted UniFrac distance metric for Bacteria, Archaea, and Eukarya. PCoA on the phylogenetic distances among samples revealing the main taxa contributing to differences in their microbial diversity **(A)**. PCoA to visualize similarities in the microbial composition **(B)**.

Using OTU count data, a PCoA of weighted UniFrac distances was conducted to explore the compositional similarities among the bacterial, archaeal, and eukaryotic communities across all distinct microhabitats (**Figure 5**). PCoA on the phylogenetic distances among samples revealed the main taxa contributing to differences in their microbial diversity. Sediment and biofilm at the interface sediment/water samples (S, BS) were characterized by the Gammaproteobacteria order RCP1-48 and Methanomassiliicoccaceae archaea. The Nitrospira genus *Leptospirillum* was distinctively contributing to community structure differences in the subaerial biofilm (BF). *Ferroplasma* archaea were differentially detected in the acid mine water inside of the gallery, whereas the genus *Thermogymnomonas* was associated to the acid water accumulating right at the entrance (WEN) and outside the gallery (WOUT). Ciliate protozoa (Spirotrichea) mainly contributed to differences in the microbial eukaryotic community of the streamer B1. Bacillariophyceae (diatoms) were found in the acid water accumulating outside the gallery (WOUT), and unclassified Opisthokonta contributed to the differences observed in the water accumulating at the entrance of the mine (WEN) (**Figure 5A**).

The PCoA reflected no clear clustering of samples, indicating the existence of very fine adaptations to environmental conditions leading to microhabitat-specific community compositions (**Figure 5B**).

The correlation between the distribution of bacterial and archaeal species and environmental factors was revealed by CCA analysis. The CCA was conducted to examine correlations between classes with a relative abundance greater than 1% in at least one sequencing library and geochemical parameters [pH, temperature, Eh, Luminance (Lx), redox potential, and conductivity] in each sample (Supplementary Figure S3). Only the environmental variables pH and Luminance showed a significant correlation (*p*-value < 0.05) with the bacterial community composition (Supplementary Figure S3A). While non-significant, the environmental variables influencing the composition of the communities of archaea and microbial eukaryotes are also displayed in Supplementary Figures S3B,C).

### 18S rRNA Gene Phylogeny Reconstruction

**FIGURE 6:**
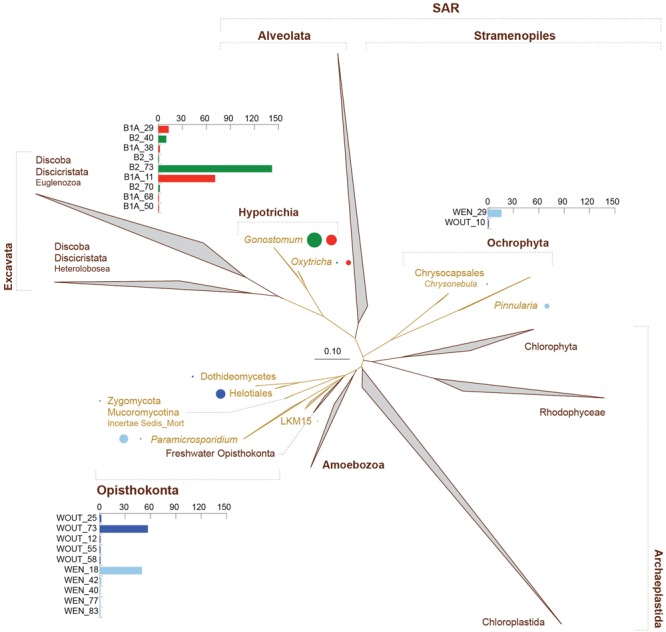
Phylogeny of 18S rRNA full-length genes recovered from biofilm (B1A and B2) and water samples (WOUT and WEN). All clades from which microbial eukaryotes had been reported in AMD ecosystems were included. The groups containing newly detected microbial signatures are highlighted in yellow. Bar graphs represent number of sequences per OPU. Bubble charts are proportional to the abundance of each specified taxa. Scale bar represents changes per site (%).

In order to obtain a quality phylogeny for the unexplored eukaryotes from Los Rueldos AMD, we performed full length 18S rRNA gene clone library analysis in parallel to next generation amplicon sequencing. Full length 18S rRNA genes were successfully amplified from samples WOUT, WEN, B1A, and B2. A total of 668 full-length 18S rRNA sequences (WOUT: 165; WEN: 189; B1A: 146, and B2: 168) were obtained and analyzed. Phylogenetic analysis of the eukaryotic sequences revealed the existence of 21 operational phylogenetic units (OPUs) distributed across the taxonomic groups Hypotrichia (ciliate protozoa), Ochrophyta (a group of photosynthetic algae), and Opisthokonta (the fungi/metazoa group, represented by microscopic fungi in the AMD environment) (**Figure 6**, yellow colored clades). Ciliate protozoa were only detected in the B1A and B2 biofilms, and were mainly represented by the genus *Gonostomum* (OPUs B1A_11 and B2_73) and *Oxytricha* (B1A_29 and B2_40). The freshwater algae *Chrysonebula* (OPU WOUT_10) and *Pinnularia* (WEN_29) were detected in the acid water accumulating outside the gallery (WOUT) and right at the entrance of the cave (WEN). Main OPUs within the Opisthokonta fungi mainly affiliated within the Ascomycota Helotiales (WOUT_73), which were detected in the water outside the gallery, and the genus *Paramicrosporidium* (WEN_18), which was found in the water at the gallery entrance. Taxa identified in lower proportions within the Opisthokonta included the Ascomycota Dothideomycetes (WOUT), Zygomycota (Mucoromycotina) (WOUT), and group LKM15 (WEN) (**Figure 6**).

### Prediction of Functional Profiles in AMD Samples

Prediction of the microbial community function was conducted using PICRUSt (Langille et al., 2013), which infers a community’s metagenome by the estimation of its gene families through the detected 16S rRNA gene signatures. PICRUSt does not allow metagenomic inference from 18S rRNA gene hypervariable region sequencing, therefore the prediction is restricted to bacterial and archaeal functions. PICRUSt has been proven to be effective for environments such as the human gut (Langille et al., 2013), for which extensive gene catalogs both from an increasing number of isolates and *in silico* genome reconstructions exist. Nevertheless, the predictions are rather sparse and generic when PICRUSt is applied using phylogenetic marker gene signatures from less known environments. Our results fit into this last category and have been included in the Supplementary Materials.

## Discussion

### Comparative Microbial Diversity

In this study, we surveyed the microbial diversity across the different microhabitats in an AMD ecosystem using high throughput sequencing of SSU rRNA phylogenetic marker genes for all domains of life. We further complemented deep sequencing of eukaryotic 18S rRNA genes with full gene length clone library analysis followed by phylogeny reconstruction. Bacteria inhabiting acidic water, sediment, biofilms, and stalactites correspond to lineages previously found in other AMDs, and affiliated to the phyla Proteobacteria, Nitrospirae, Actinobacteria, Firmicutes, and Acidobacteria (Xie et al., 2007; Tan et al., 2009; García-Moyano et al., 2012; Méndez-García et al., 2015).

Cyanobacteria predominated in water samples under the influence of light (WOUT and WEN). The first cultivation-independent detection of this phylum in AMD was firstly reported by González-Toril et al. (2003) in the Tinto river. The presence of eukaryotes was further reported in other AMDs, e.g., in Zn mine tailings in Sepetiba Bay, Brazil (Almeida et al., 2009), or in a pH 2.8 AMD from Xiang Mountain, China (Hao et al., 2012). Cyanobacteria are photosynthetic microorganisms able to fix nitrogen, therefore contributing importantly to C and N cycling in the habitat. The proportion of these microorganisms was drastically reduced inside the gallery of Los Rueldos, where Proteobacteria predominated, a feature shared with most of the described AMD environments (Méndez-García et al., 2015).

Phyla AD3 and Acidobacteria were mainly detected in sediments in the Los Rueldos system. AD3 bacteria had been detected in biofilms thriving in Los Rueldos AMD (Méndez-García et al., 2014) and, to the best of the authors knowledge, AD3 soil bacteria have not been previously detected in other AMD environments. Acidobacteria were also found in soils, and had been formerly detected in AMDs sediments (Ward et al., 2009) and acidic biofilms (Méndez-García et al., 2014).

Extreme pH conditions select for a lower microbial diversity, while moderate pH conditions allow colonization by a wider variety of taxa (Baker and Banfield, 2003; Johnson and Hallberg, 2003). Thus, the higher bacterial diversity detected in the stalactite could be attributed to its higher pH (∼ 6.5), as compared to the rest of the samples. The rainwater draining into the gallery would likely drag microorganisms to the cave ceiling and through stalactites, resulting in a higher species richness. Interestingly, a few bacterial phyla were only detected in the stalactite, namely Chloroflexi, Chlorobi, Bacteroidetes, and candidate phylum WPS-2. Chloroflexi bacteria are common in sediments, with a predicted important role in carbon cycling (Hug et al., 2013), and had been detected in AMD environments before (García-Moyano et al., 2015). Chlorobi had been detected in a soil associated to mine drainage (Pereira et al., 2014) and acid mine water samples (Volant et al., 2014). Green sulfur bacteria (Chlorobiaceae), together with the family Ignavibacteriaceae, belong to the phylum Chlorobi. Chlorobiaceae are obligately anaerobic phototrophs, whereas Ignavibacteria are non-photosynthetic chemoheterotrophs. The presence of genetic signatures affiliating within this phylum in the stalactite might suggest the possibility of an aero-tolerant or aerobic lifestyle in some close bacterial relatives. Down to the genus level, betaproteobacteria of the genus *Gallionella* was uniquely detected in the stalactite and absent in the rest of the samples with lower pH, in concordance to its niche as neutrophilic iron oxidizer (Bruneel et al., 2006; Bertin et al., 2011). Genetic signatures for WPS-2 bacteria had been previously detected in a pH neutral mine drainage in Brazil (Pereira et al., 2014). Members of Bacteroidetes had been found in an AMD in the high Artic (Norway) (García-Moyano et al., 2015), but they are uncommon in oxidized environments (Donaldson et al., 2016).

Firmicutes of the class Clostridia were detected in slightly higher proportion in the subaerial biofilm growing at the interface rock/water (BF). Genera belonging to this class, e.g., *Sulfobacillus* spp., have been extensively reported in subaerial biofilms (Justice et al., 2014). Genetic signatures with high similarity to candidate division *Ca*. Saccharibacteria bacteria were detected mostly in the acid water, and had been identified in the thick stratified B1AB and suboxic B2 biofilms thriving in Los Rueldos AMD (Méndez-García et al., 2014). The biogeography of these bacteria and function in AMD habitats remains to be elucidated.

The archaeal lineages detected were similar to those reported in previous studies (Bond et al., 2000b; Sánchez-Andrea et al., 2011; Yelton et al., 2013). We were able to identify two of the most frequently AMD-associated genera within the Thermoplasmatales euryarchaeota: *Ferroplasma* and *Thermogymnomonas*. *Ferroplasma* spp. are capable of oxidizing Fe and organic matter, and grow anaerobically via Fe respiration (Edwards et al., 2000; Dopson et al., 2004). Our current knowledge on the physiology of *Thermogymnomonas* spp. is based on observations on the sole cultivated species *T. acidicola*, isolated from a solfataric field in Japan (Itoh et al., 2007). The species is obligately aerobic and heterotrophic. In Los Rueldos, *Thermogymnomonas* was detected in high proportions in all samples, with chemical characteristics compatible with its niche as a heterotrophic obligate aerobe (Itoh et al., 2007). Signatures for the genus *Ferroplasma* where only detected in the acid water (WIN) and in the stalactite (ST), probably indicating a higher content of reduced iron species in these compartments.

Interestingly, the Micrarchaea candidate group was present in proportions higher than 1% only in the acid water (WIN). This group remains to be characterized, and seems to represent a separate phylum within the superphylum DPANN (Rinke et al., 2013; Castelle et al., 2015). Micrarchaea, mainly represented by the ARMAN candidate genus “Micrarchaeum acidophilum” (Baker et al., 2006, 2010), had been previously detected in acid water (e.g., Hua et al., 2015) and biofilm microhabitats from AMD ecosystems (e.g., Méndez-García et al., 2014).

The bacteria and archaea identified in this study have been reported to be able to oxidize Fe and/or S (*Leptospirillum* spp., *Acidithiobacillus* spp., *Ferroplasma* spp., *Acidimicrobium* spp., *Thiomonas* spp., and *Gallionella* spp.), to reduce Fe and/or S (*Acidithiobacillus* spp., *Acidiphilium* spp., *Acidimicrobium* spp., and *Ferroplasma* spp.), and to importantly contribute to carbon cycling (*Acidiphilium* spp. and *Acidobacterium* spp.) and N fixation (e.g., *Leptospirillum* spp.) (Méndez-García et al., 2015).

Microbial eukaryotes were analyzed by two complementary approaches, next generation sequencing of the 18S rRNA gene hypervariable region V9 and 18S rRNA full-length gene clone library survey. Notably, both provided comparable results and allowed to recover the most dominant members of the community in a reproducible manner, with the exception of that 18S rRNA gene signatures in B2 could only be obtained through clone library analysis. The highest diversity of eukaryotes in Los Rueldos samples was found in the acid water accumulating outside of the gallery, which received the major influence of the sunlight. According to their taxonomic affiliation and their presence only in the outer region of the drainage, we can infer that most of these eukaryotes are phototrophic. Most detected taxa had been detected in open-air AMD systems, such as the Tinto river, where photosynthesis is the primary activity implicated in carbon fixation (González-Toril et al., 2003).

The main groups of eukaryotes detected in Los Rueldos samples belong to the supergroup SAR and to the Opisthokonta clade. Eukaryotes present in the acid water at the pond outside and at the entrance of the gallery (WOUT and WEN) mainly affiliated within the Stramenopiles (SAR) and Fungi (Opisthokonta) groups. The detected Stramenopiles belonged to Diatomea and members of the class Chrysophyceae. The diatom *Pinnularia* (Ochrophyta, Stramenopiles) was estimated to be more abundant in WEN, whereas *Chrysonebula* was detected in WOUT. *Pinnularia*, which is a known acidophilic diatom, had been found in the conspicuous biofilms thriving at the Tinto river (Amaral-Zettler, 2012; Luís et al., 2012). Chrysophyceae is a large class of freshwater algae, and was reported to be a dominant member in the Tinto river AMD (Amaral Zettler et al., 2002). To the best of the authors knowledge, this is the first time that the genus *Chrysonebula* was detected in a low pH drainage with toxic concentrations of metallic species. Fungi detected in water samples mainly belonged to the groups Pezizomycotina, LKM11, and Nucletmycea. Fungi were the dominant group in Carnoulès sediments and in the Richmond Mine (Iron Mountain, Los Angeles, CA, United States) biofilms (Baker et al., 2004; Volant et al., 2016). The environmental fungal clade LKM11 belongs to a group of fungi located near the phylogenetic root of the fungal kingdom, Rozellomycota. The clade is primarily comprised by Rozellida, a group of parasites with algal and fungal hosts (Lara et al., 2010; Masquelier et al., 2010; Auld et al., 2016). Phylogeny reconstruction indicated that the LKM11 group shared high similarity with *Paramicrosporidium* fungi, which are endonuclear parasites of free-living amoebae (Corsaro et al., 2014a,b). Interestingly, the recovery of amoebae sequences was markedly lower (<1% in all samples), which could be explained by a ratio of parasite numbers relative to host higher than 1, by the presence of hosts other than amoebae, or by the existence of uncharacterized free-living *Paramicrosporidium* taxa with 18S rRNA gene sequences similar to those of the parasite representatives.

SAR Alveolata ciliates were the predominant eukaryotes detected in the uppermost oxic (B1A) and hypoxic (B2) biofilm samples, with the genera Hypotrichia *Gonostomun* and *Oxytricha* (phylum Ciliophora, class Spirotrichea, subclass Hypotrichia) detected in both formations. *Gonostomum* spp. are widely distributed in terrestrial and limnetic habitats (Berger, 1999; Vd’ačný and Tirjaková, 2006; Kamra et al., 2008). Their presence at low pH and in biofilm samples is novel, and may reflect fine adaptation to very steep environmental gradients. The ciliate *Oxytricha* had been detected previously in AMD environments (Hao et al., 2010; González-Toril et al., 2011; Weisse et al., 2013). Ciliates and other protozoa are major predators of bacteria, and provide an important trophic link in aquatic habitats such as stream biofilms (McGinness and Johnson, 1992; Baker et al., 2004; Dopheide et al., 2009; Volant et al., 2016). Ciliate species (*Oxytrichia*) occurred also in high numbers in acid streamers in the underground uranium mine Königstein (Germany) (Zirnstein et al., 2012). Baker et al. (2009) analyzed the eukaryotic diversity in biofilms growing in metal-rich underground AMD solutions at the Richmond mine and reported a limited diversity of ciliates. In contrast, their results indicated a dominance of fungi (68%), acidophilic protist clade (APC), and Heterolobosea.

### Microbial Ecology Model at Los Rueldos

Previous studies in streamer and mat biofilms at Los Rueldos revealed a metabolically stratified active ecosystem, indicating that archaea and bacteria thriving in the AMD-air interface are more active in terms of iron, nitrogen, and hydrogen usage. The suboxic communities appeared to have major roles in sulfur and carbon transformations, with dissolved oxygen being the primary force driving metabolic differences among strata (Méndez-García et al., 2014).

González-Toril et al. (2011) suggested that photosynthetic eukaryotes play a fundamental role in the formation of atmospheric oxygen, which could favor the aerobic oxidation of iron and sulfur. This could be also applicable to Los Rueldos AMD. Two non-photosynthetic eukaryotic lineages (ciliates and fungi) were also detected. Ciliates, a dominant group in B1A and also present in B2, can impact the abundance of bacteria and archaea, and therefore, the community composition and its function. Fungi could otherwise contribute to the decomposition of organic compounds and have an important role in carbon cycling.

**FIGURE 7:**
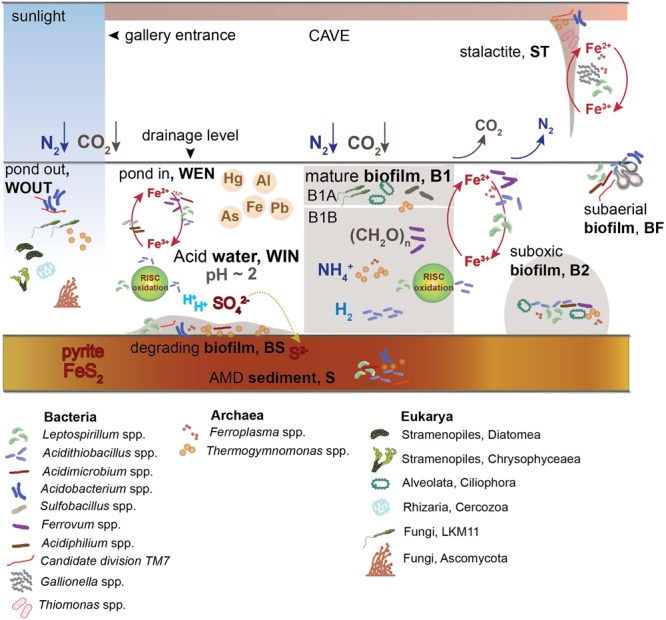
Summarized model integrating diversity and predicted function in Los Rueldos microhabitats (mineral, water, and biofilm). *Leptospirillum* spp., Acidithiobacillales, and Betaproteobacteria are probable drivers of iron and sulfur oxidation in mineral (S and ST), water (WOUT, WEN, and WIN), and biofilm samples (BS, B1A, B1B, B2, and BF). The distribution of the neutrophilic iron oxidizer *Gallionella* is restricted to the stalactite ST. Carbon fixers are predictively photosynthetic in water ponds outside the gallery and are mainly represented by Cyanobacteria. *Leptospirillum* spp. are suggested to perform inorganic carbon and nitrogen fixation in all microhabitats inside of the cave. Decomposition of organic matter in the drainage accumulating outside of the gallery (WEN) would support the life of heterotrophic community members (Fungi, Protozoa, Proteobacteria, and Archaea) in this compartment. The extracellular polymeric matrix of the biofilms would constitute the main carbon source inside of the gallery, supporting the heterotrophic lifestyle of most abundant microbial groups present in the cave (Ciliates, Proteobacteria, and Archaea). The stagnant nature of the AMD would favor the existance of marked microhabitats along the drainage. Biofilm ciliates could have an impact on the population structure of bacterial members of the community, and the presence of different genera in distinct biofilm strata likely relates to differential bacterial/archaeal community composition, suggesting adaptation to distinctive environmental variables (oxic/hypoxic, water/mineral fraction).

The functional prediction analysis suggested a dependence of the community function on the pH (Supplementary Figure S4C), which reflects the adaptation of the microbial communities to the extreme condition. Prediction of community function based on previously described metabolic capabilities of the detected microorganisms revealed *Leptospirillum* spp. as main drivers of iron oxidation in the subaerial (BF) and degrading (BS) biofilms, the latter collected at the interface biofilm/sediment. This bacterium can also fix inorganic C and N (Tyson et al., 2004; Aliaga Goltsman et al., 2009). The presence of Fe and/or S oxidizers from the *Acidithiobacillus* genus was >2% in all samples. Acidithiobacillales from the group RCP1-48, detected in high proportions in BF, BS, and WEN, could also have roles in Fe/S oxidation, but in spite of having been reported in other AMD habitats (e.g., Sánchez-Andrea et al., 2011), their metabolic features remain uncharacterized. Betaproteobacteria, dominating in BF and WIN, were not classified at lower taxonomic levels, and therefore their roles in the community remain unknown. Previously detected Betaproteobacteria in Los Rueldos were phylogenetically related to *Ferrovum* spp. and represented high proportions in B1AB and B2 biofilms (Méndez-García et al., 2014). *Fv. myxofaciens* is an iron-oxidizing acidophilic bacterium, able to produce copious amounts of extracellular polymeric substances (Johnson et al., 2014), therefore relatives to this genus could account for a fraction in BF, but this is highly speculative, and taxonomy-specific PCR or phylogeny reconstruction of full-length 16S rRNA genes would be needed to further resolve the phylogeny of the detected Betaproteobacteria (**Figure 7**). A genus affiliating within the Xanthomonadaceae was found in most samples analyzed, and might include heterotrophic members, a metabolic trait that characterizes AMD-related Xanthomonadaceae (Johnson and Hallberg, 2003), and that was the defining metabolic feature of the low pH oxic and suboxic B1A and B2 biofilms present in Los Rueldos AMD (Méndez-García et al., 2014) (**Figure 7**).

From a systems microbiology perspective, the compartment represented by WOUT is predicted to have a major influence in metabolite trafficking. Water samples are predicted to harbor microorganisms with genomic contents enriched in coding sequences for chaperones, folding catalysts, and DNA repair, as they are more exposed to the extreme conditions than biofilm samples. Biofilms showed, as expected, a higher content of signatures for chemotaxis. Amino acid metabolism signatures were evenly distributed, with hypoxic compartments (B1B, S, and B2) more implicated in degradation, especially of amino acid lysine. Oxic strata (B1A and BF) are predicted to release energy through the TCA cycle. Signatures related to the glyoxylate cycle, by which organic compounds (i.e., fatty acids) that are degraded to acetyl-CoA can be used for biosynthesis (Yelton et al., 2013), were more abundant in WOUT/WEN and B2, suggesting the existence of anabolic pathways for carbohydrate biosynthesis in the biofilm in the presence of acetate, oxidation of accumulated organic matter in WOUT and proximities of WEN, and degradation of exopolysaccharides in B2.

Overall, our survey on the microbial diversity pertaining to the three domains of life within Los Rueldos AMD revealed a unique diversity in the different microhabitats identified in the ecosystem, and a similarity of predicted adaptive responses to prevailing extreme conditions in spite of dissimilarities in the composition on the discrete microbial communities.

## Author Contributions

Field work and data analysis were carried out by CM-G and VM. RG-G contributed to statistical analysis. CM-G, VM, JS, and AP wrote and revised the paper. All authors have read and approved this manuscript.

## Conflict of Interest Statement

The authors declare that the research was conducted in the absence of any commercial or financial relationships that could be construed as a potential conflict of interest.
