# Supplementary material for: Bacterial, Archaeal, and Eukaryotic Diversity across Distinct Microhabitats in an Acid Mine Drainage
Source: Front Microbiol. 2017 Sep 12;8:1756. doi: 10.3389/fmicb.2017.01756 (PMC5600952; doi:10.3389/fmicb.2017.01756)
Supplement: Supplementary file 2 [file Image_1.pdf]

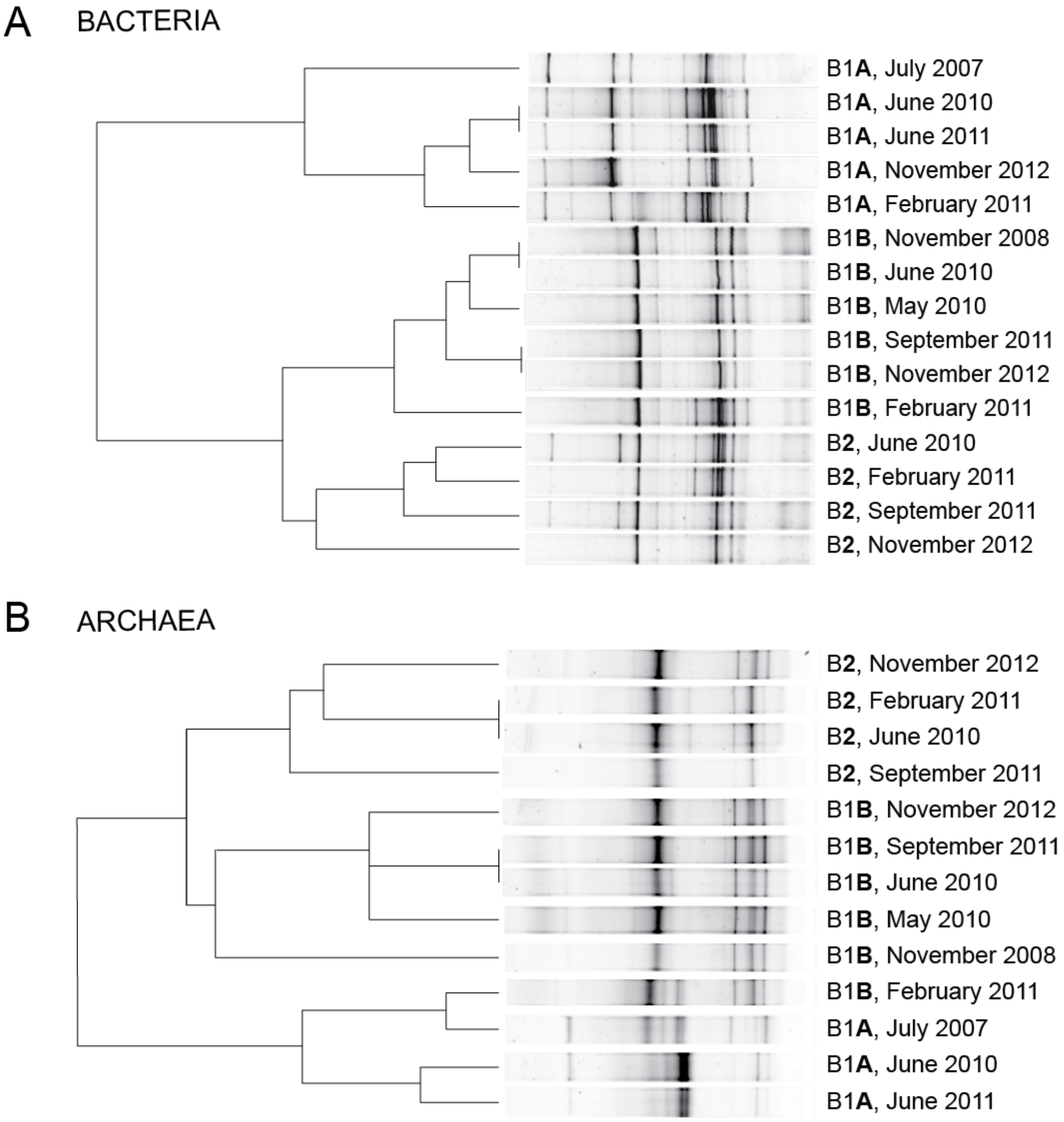

Supplementary Figure 1. UPGMA clustering of DGGE profiles for bacterial sequences in sampling efforts of B1A, B1B and B2 biofilms in the period 2007-2012.
