# Supplementary material for: Bacterial, Archaeal, and Eukaryotic Diversity across Distinct Microhabitats in an Acid Mine Drainage
Source: Front Microbiol. 2017 Sep 12;8:1756. doi: 10.3389/fmicb.2017.01756 (PMC5600952; doi:10.3389/fmicb.2017.01756)
Supplement: Supplementary file 3 [file Image_2.pdf]

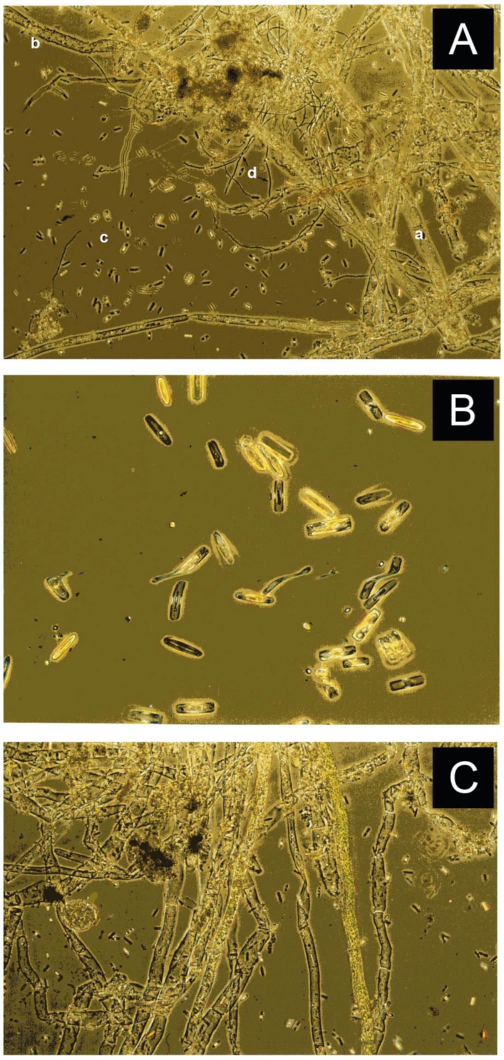

Supplementary Figure 2. Images of phase-contrast microscope of Los Rueldos eukaryotes. **(A)** [a], [b], filamentous algae; [c] diatoms; [d] cyanobacteria (40X). **(B)** Detail on the different morphotypes of Los Rueldos diatoms (100X). **(C)** Image displaying low abundant protozoans (likely from the class *Spirotrichea*, which includes ciliate protozoans) in open-air water samples (40X).
