# Supplementary material for: Bacterial, Archaeal, and Eukaryotic Diversity across Distinct Microhabitats in an Acid Mine Drainage
Source: Front Microbiol. 2017 Sep 12;8:1756. doi: 10.3389/fmicb.2017.01756 (PMC5600952; doi:10.3389/fmicb.2017.01756)
Supplement: Supplementary file 4 [file Image_3.pdf]

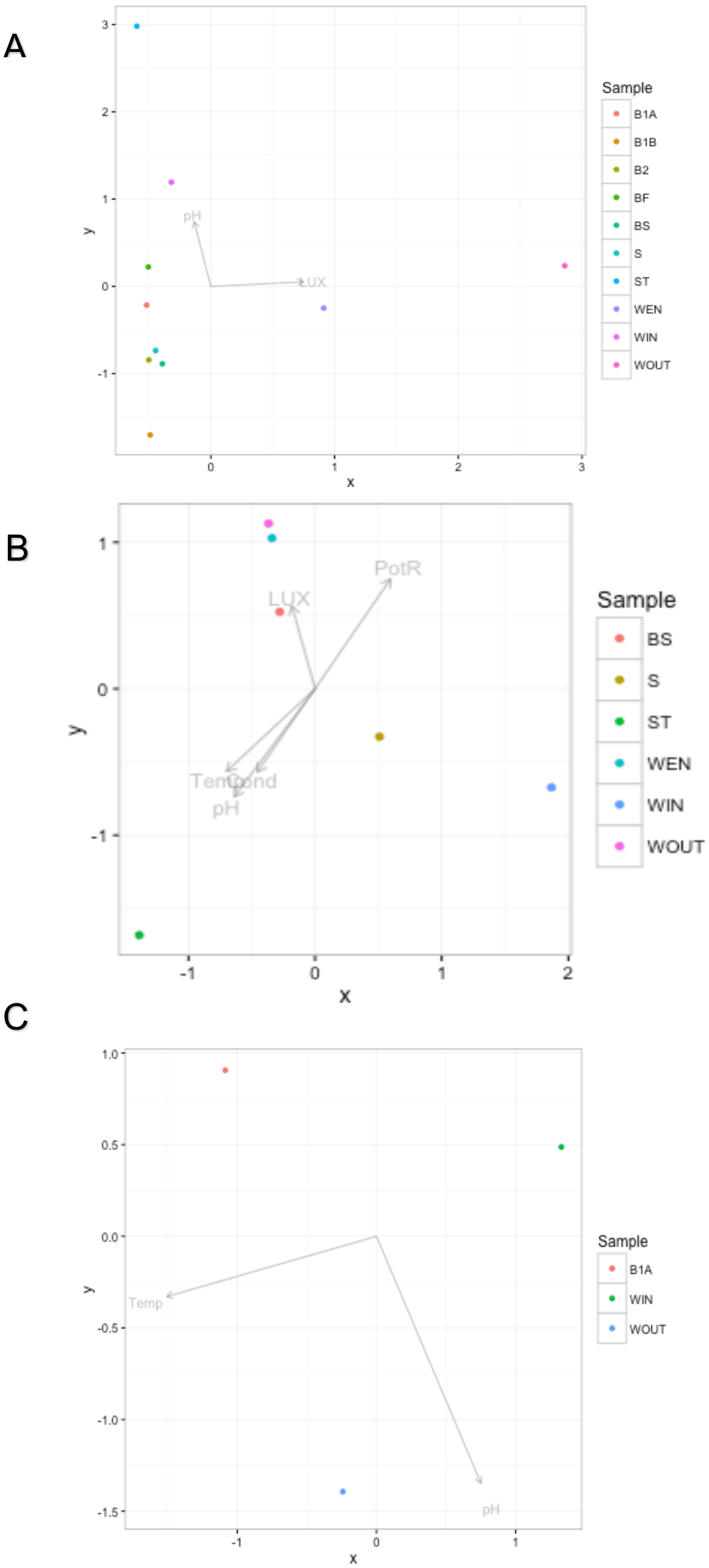

Supplementary Figure 3. Ordination diagrams from canonical correspondence analysis (CCA) of bacterial **(A)**, archaeal **(B)** and eukarya **(C)** abundances, and geochemical parameters. Arrows indicate the direction and magnitude of geochemical parameters associated with community structures. Each sample is represented by colored circles according to the collection sites. Abbreviations: PotR, Potential Redox; LUX, Luminance; Temp, Temperature; Cond, Conductivity.
