# Supplementary material for: Bacterial, Archaeal, and Eukaryotic Diversity across Distinct Microhabitats in an Acid Mine Drainage
Source: Front Microbiol. 2017 Sep 12;8:1756. doi: 10.3389/fmicb.2017.01756 (PMC5600952; doi:10.3389/fmicb.2017.01756)
Supplement: Supplementary file 5 [file Image_4.pdf]

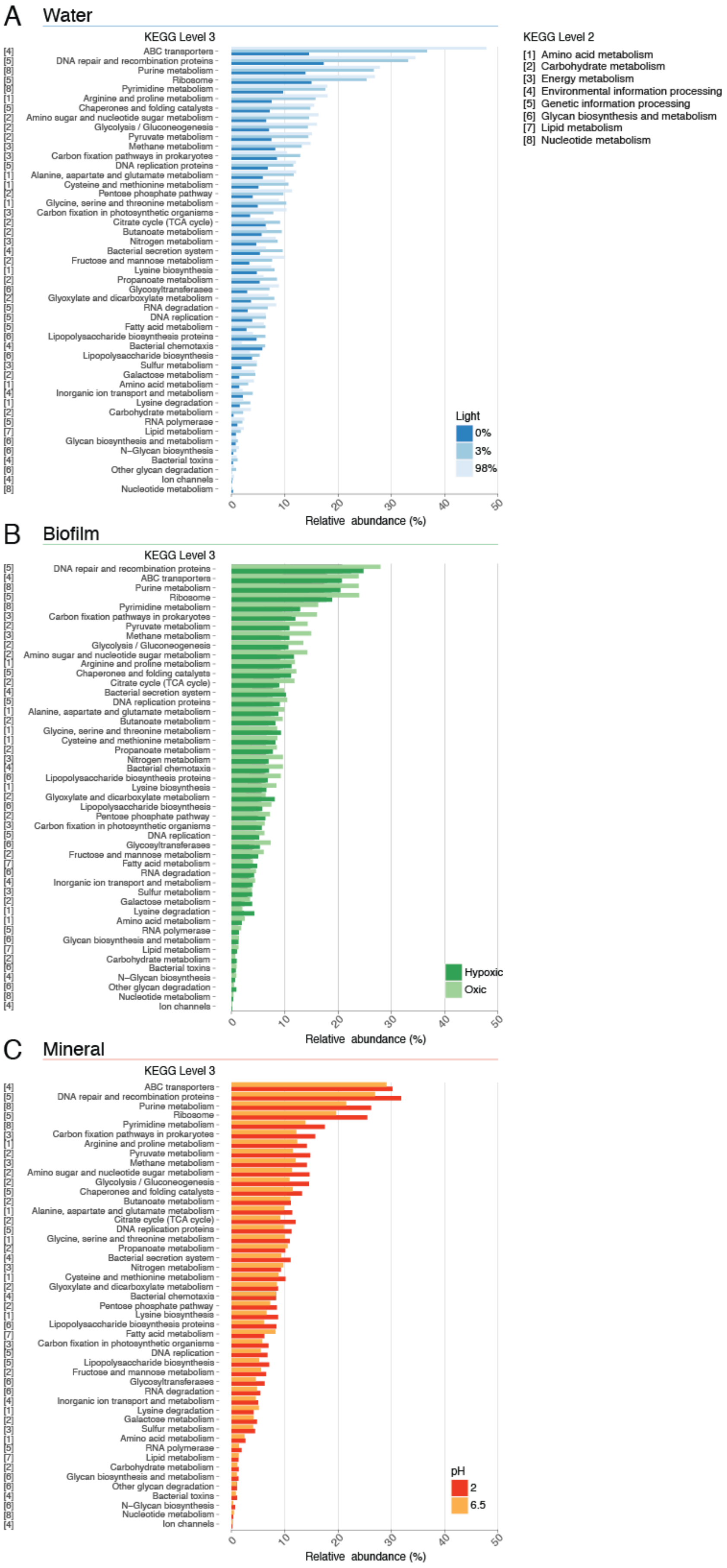
A Water
KEGG Level 3
[4] ABC transporters
[5] DNA repair and recombination proteins
[8] Purine metabolism
[5] Ribosome
[8] Pyrimidine metabolism
[1] Arginine and proline metabolism
[5] Chaperones and folding catalysts
[2] Amino sugar and nucleotide sugar metabolism
[2] Glycolysis / Gluconeogenesis
[2] Pyruvate metabolism
[3] Methane metabolism
[3] Carbon fixation pathways in prokaryotes
[5] DNA replication proteins
[1] Alanine, aspartate and glutamate metabolism
[1] Cysteine and methionine metabolism
[2] Pentose phosphate pathway
[1] Glycine, serine and threonine metabolism
[3] Carbon fixation in photosynthetic organisms
[2] Citrate cycle (TCA cycle)
[2] Butanoate metabolism
[3] Nitrogen metabolism
[4] Bacterial secretion system
[2] Fructose and mannose metabolism
[1] Lysine biosynthesis
[2] Propanoate metabolism
[6] Glycosyltransferases
[2] Glyoxylate and dicarboxylate metabolism
[5] RNA degradation
[5] DNA replication
[5] Fatty acid metabolism
[6] Lipopolysaccharide biosynthesis proteins
[4] Bacterial chemotaxis
[6] Lipopolysaccharide biosynthesis
[3] Sulfur metabolism
[2] Galactose metabolism
[1] Amino acid metabolism
[4] Inorganic ion transport and metabolism
[1] Lysine degradation
[2] Carbohydrate metabolism
[5] RNA polymerase
[7] Lipid metabolism
[6] Glycan biosynthesis and metabolism
[6] N-Glycan biosynthesis
[4] Bacterial toxins
[6] Other glycan degradation
[4] Ion channels
[8] Nucleotide metabolism
0 10 20 30 40 50
Relative abundance (%)
Light
0%
3%
98%
KEGG Level 2
[1] Amino acid metabolism
[2] Carbohydrate metabolism
[3] Energy metabolism
[4] Environmental information processing
[5] Genetic information processing
[6] Glycan biosynthesis and metabolism
[7] Lipid metabolism
[8] Nucleotide metabolism
B Biofilm
KEGG Level 3
[5] DNA repair and recombination proteins
[4] ABC transporters
[8] Purine metabolism
[5] Ribosome
[8] Pyrimidine metabolism
[3] Carbon fixation pathways in prokaryotes
[2] Pyruvate metabolism
[3] Methane metabolism
[2] Glycolysis / Gluconeogenesis
[2] Amino sugar and nucleotide sugar metabolism
[1] Arginine and proline metabolism
[5] Chaperones and folding catalysts
[2] Citrate cycle (TCA cycle)
[4] Bacterial secretion system
[5] DNA replication proteins
[1] Alanine, aspartate and glutamate metabolism
[2] Butanoate metabolism
[1] Glycine, serine and threonine metabolism
[1] Cysteine and methionine metabolism
[2] Propanoate metabolism
[3] Nitrogen metabolism
[4] Bacterial chemotaxis
[6] Lipopolysaccharide biosynthesis proteins
[1] Lysine biosynthesis
[2] Glyoxylate and dicarboxylate metabolism
[6] Lipopolysaccharide biosynthesis
[2] Pentose phosphate pathway
[3] Carbon fixation in photosynthetic organisms
[5] DNA replication
[6] Glycosyltransferases
[2] Fructose and mannose metabolism
[7] Fatty acid metabolism
[5] RNA degradation
[4] Inorganic ion transport and metabolism
[3] Sulfur metabolism
[2] Galactose metabolism
[1] Lysine degradation
[1] Amino acid metabolism
[5] RNA polymerase
[6] Glycan biosynthesis and metabolism
[7] Lipid metabolism
[2] Carbohydrate metabolism
[4] Bacterial toxins
[6] N-Glycan biosynthesis
[6] Other glycan degradation
[8] Nucleotide metabolism
[4] Ion channels
0 10 20 30 40 50
Relative abundance (%)
Hypoxic
Oxic
C Mineral
KEGG Level 3
[4] ABC transporters
[5] DNA repair and recombination proteins
[8] Purine metabolism
[5] Ribosome
[8] Pyrimidine metabolism
[3] Carbon fixation pathways in prokaryotes
[1] Arginine and proline metabolism
[2] Pyruvate metabolism
[3] Methane metabolism
[2] Amino sugar and nucleotide sugar metabolism
[2] Glycolysis / Gluconeogenesis
[5] Chaperones and folding catalysts
[2] Butanoate metabolism
[1] Alanine, aspartate and glutamate metabolism
[2] Citrate cycle (TCA cycle)
[5] DNA replication proteins
[1] Glycine, serine and threonine metabolism
[2] Propanoate metabolism
[4] Bacterial secretion system
[3] Nitrogen metabolism
[1] Cysteine and methionine metabolism
[2] Glyoxylate and dicarboxylate metabolism
[4] Bacterial chemotaxis
[2] Pentose phosphate pathway
[1] Lysine biosynthesis
[6] Lipopolysaccharide biosynthesis proteins
[7] Fatty acid metabolism
[3] Carbon fixation in photosynthetic organisms
[5] DNA replication
[6] Lipopolysaccharide biosynthesis
[2] Fructose and mannose metabolism
[6] Glycosyltransferases
[5] RNA degradation
[4] Inorganic ion transport and metabolism
[1] Lysine degradation
[2] Galactose metabolism
[3] Sulfur metabolism
[1] Amino acid metabolism
[5] RNA polymerase
[7] Lipid metabolism
[2] Carbohydrate metabolism
[6] Glycan biosynthesis and metabolism
[6] Other glycan degradation
[4] Bacterial toxins
[6] N-Glycan biosynthesis
[8] Nucleotide metabolism
[4] Ion channels
0 10 20 30 40 50
Relative abundance (%)
pH
2
6.5

Supplementary Figure 4. Bar graphs showing the relative abundances of genomic signatures predicted by PICRUSt within KEGG Level 3 categories among samples per microhabitat considered (water [A], biofilm [B], mineral fraction [C]), and geochemical variables (light [0, 3, or 98%] for water samples in shades of blue, depth [hypoxic, oxic] for biofilm samples in green colors, and pH [2 and 6.5] for mineral fraction samples in red tones). KEGG Level 2 categories are displayed on the y axis, and the corresponding legend is presented on the right.
