# Supplementary material for: Bacterial, Archaeal, and Eukaryotic Diversity across Distinct Microhabitats in an Acid Mine Drainage
Source: Front Microbiol. 2017 Sep 12;8:1756. doi: 10.3389/fmicb.2017.01756 (PMC5600952; doi:10.3389/fmicb.2017.01756)
Supplement: Supplementary file 6 [file Data_Sheet_2.pdf]

Supplementary Material

Bacterial, archaeal, and eukaryotic diversity across distinct microhabitats in an acid mine drainage

Victoria Mesa[1,2*], Jose L. R. Gallego[3], Ricardo González-Gil[4], Béatrice Lauga[5], Jesús Sánchez[1], Celia Méndez-García[6†] and Ana I. Peláez[1†]

*** Correspondence:** Corresponding Author: victoriamesa@gmail.com

1 Supplementary Data

Prediction of functional profiles in AMD samples

PICRUSt was used as a predictive exploratory tool to obtain an overview of the genomic and metabolic features in Los Rueldos AMD habitat. The mean NSTI was similar (0.25 ± 0.08 s.d. bacterial data and 0.12 ± 0.08 s.d. archaeal data) to that reported for hypersaline mat microbiome (0.23 ± 0.07 s.d.) (Langille et al., 2013). The analysis was performed by grouping samples into biofilm (BF, B1A, B1B, B2, BS), mineral fractions (S, ST), and water (WOUT, WEN, WIN) as identifiable microhabitats in Los Rueldos AMD system, and main physicochemical variables accounting for differences in each microhabitat were considered (percentage of outside light in water samples, depth in biofilm samples, and pH in mineral fraction samples). Previously acquired data on B1 streamer and B2 submerged mat was included for a complete comparative analysis (Méndez-García et al., 2014). The considered KEGG functional categories were related to biogeochemical cycling of elements (C, N, S, Fe, H), cell function (e.g., DNA replication and transcription), and/or or adaptation to extreme conditions (e.g., chaperones and folding catalysts).

Environmental information processing. This KEGG category was the most represented among the eight predicted by PICRUSt (Figure S4). Overall, water samples (WOUT, WEN, WIN) were predicted to be more involved in environmental information processing ($p > 0.05$), with KEGG Level 3 category “ABC transporters” contributing mainly to the observed differences. Bacteria present in WOUT (98% sunlight) were predicted to harbor a higher genomic content of transporters and ion channels, followed by WEN (3% sunlight), and WIN (complete darkness). There existed a trend towards enrichment of bacterial secretion and transport of inorganic compounds genomic signatures in mineral fraction samples (S, ST), whereas bacterial chemotaxis was mostly represented in biofilm samples (B1A, B1B, B2, BF, BS), especially in the oxic strata (Figure S4).

Genetic information processing. There were no significant differences found among all microhabitats considered in their predicted genomic signatures related to genetic information processing ($p > 0.05$), the second major represented KEGG category. There was a trend towards a higher presence of genomic signatures for RNA degradation proteins, chaperones and folding catalysts, DNA replication, recombination and repair, RNA polymerases, and ribosomal compounds in water samples, with WOUT contributing mainly to the observed tendency (Figure S4).

Nucleotide metabolism. Nucleotide metabolism constituted the third category in prediction assignments among all eight identified. Purine metabolism signatures were more abundant than pyrimidine metabolism signatures ($p > 0.05$), with WOUT, B1A, BF, and S contributing mainly to the observed variance (Figure S4).

Amino acid metabolism. Predicted signatures for the metabolism of the amino acids arginine and proline were the most abundant in all samples, followed by those for amino acids alanine, aspartate, glutamate, glycine, serine, threonine, cysteine, methionine, and lysine. Differences among microhabitats were not significant, but within water samples, amino acid metabolism signatures were noticeably more abundant in samples with some influence of the sunlight (WOUT, WEN). This trend was also observed in oxic biofilm samples (B1A, BF), with the particular exception of lysine degradation, for which signatures were more abundant in hypoxic biofilms (B1B, B2, BS), and in mineral fraction samples (S, ST) ($p > 0.05$) (Figure S4).

Carbohydrate metabolism. Signatures related with glycolysis, gluconeogenesis, and pyruvate, glyoxylate, dicarboxylate, or galactose metabolism displayed no significant differences among microhabitats considered. Nevertheless, signatures for the glyoxylate pathway prevailed in WOUT, WEN water samples, and in hypoxic biofilms ($p < 0.05$). A trend towards enrichment of genomic signatures for TCA cycle and butanoate/propanoate metabolism was observed in oxic biofilm samples and mineral fractions. Signatures for metabolism of fructose, mannose, amino- and nucleotide sugars, as well as for the pentose phosphate pathway, were higher in water samples (primarily WOUT and WEN) (Figure S4).

Energy metabolism. Predicted genomic signatures for carbon fixation, nitrogen and methane metabolism were non-significantly enriched in oxic biofilm samples ($p > 0.05$), whereas signatures for carbon fixation in photosynthetic microorganisms were more abundant in WOUT and WEN water samples ($p > 0.05$). Sulfur metabolism signatures were found prevalently in water samples at the entrance of the gallery, but differences were non-significant among microhabitats (Figure S4).

Glycan biosynthesis and metabolism. Biofilm samples (especially oxic strata) were found to harbor a higher abundance of signatures for lipopolysaccharide biosynthesis and glycosyltransferases ($p > 0.05$). Among the three microhabitats considered, mineral fraction samples displayed the lowest predicted genomic content associated to polysaccharide metabolism (Figure S4).

Lipid metabolism. Signatures for fatty acid metabolism were comparatively higher in mineral fraction samples under higher pH, followed by biofilm (mainly hypoxic) and water samples (chiefly WOUT, WEN) ($p > 0.05$) (Figure S4).
